# Commissioning an Elekta Versa HD linear accelerator

**DOI:** 10.1120/jacmp.v17i1.5799

**Published:** 2016-01-08

**Authors:** Ganesh Narayanasamy, Daniel Saenz, Wilbert Cruz, Chul S. Ha, Niko Papanikolaou, Sotirios Stathakis

**Affiliations:** ^1^ Department of Radiation Oncology University of Texas Health Science Center at San Antonio San Antonio TX; ^2^ Department of Radiation Oncology University of Arkansas for Medical Sciences Little Rock AR; ^3^ Landauer Medical Physics Glenwood IL USA

**Keywords:** commissioning, Versa HD, linac, dosimetry, flattening filter‐free (FFF)

## Abstract

The purpose of this study is to report the dosimetric aspects of commissioning performed on an Elekta Versa HD linear accelerator (linac) with high‐dose‐rate flattening filter‐free (FFF) photon modes and electron modes. Acceptance and commissioning was performed on the Elekta Versa HD linac with five photon energies (6 MV, 10 MV, 18 MV, 6 MV FFF, 10 MV FFF), four electron energies (6 MeV, 9 MeV, 12 MeV, 15 MeV) and 160‐leaf (5 mm wide) multileaf collimators (MLCs). Mechanical and dosimetric data were measured and evaluated. The measurements include percent depth doses (PDDs), in‐plane and cross‐plane profiles, head scatter factor ($S_{c}$), relative photon output factors ($S_{\text{cp}}$), universal wedge transmission factor, MLC transmission factors, and electron cone factors. Gantry, collimator, and couch isocentricity measurements were within 1 mm, 0.7 mm, and 0.7 mm diameter, respectively. The PDDs of 6 MV FFF and 10 MV FFF beams show deeper $d_{\text{max}}$ and steeper falloff with depth than the corresponding flattened beams. While flatness values of 6 MV FFF and 10 MV FFF normalized profiles were expectedly higher than the corresponding flattened beams, the symmetry values were almost identical. The cross‐plane penumbra values were higher than the in‐plane penumbra values for all the energies. The MLC transmission values were 0.5%, 0.6%, and 0.6% for 6 MV, 10 MV, and 18 MV photon beams, respectively. The electron PDDs, profiles, and cone factors agree well with the literature. The outcome of radiation treatment is directly related to the accuracy in the dose modeled in the treatment planning system, which is based on the commissioned data. Commissioning data provided us a valuable insight into the dosimetric characteristics of the beam. This set of commissioning data can provide comparison data to others performing Versa HD commissioning, thereby improving patient safety.

PACS number(s): 87.56.bd

## I. INTRODUCTION

Versa HD is a new class of linear accelerators released by Elekta (Elekta Oncology Systems, Crawley, UK). It can deliver flattened photon beams, flattening filter‐free (FFF) photon beams, as well as electron beams. The dose rates can go up to 1400 MU/min for 6 MV FFF and 2400 MU/min for 10 MV FFF beams. Although Versa HD has been adopted in many clinics around the world, there is a paucity of information about the acceptance and commissioning tests involved, increasing the burden on physicists. In this study, commissioning data were measured and tabulated to provide information. The main goal of this work is to generate a set of technical guidelines that may assist other institutions performing a Versa HD commissioning. End‐to‐end testing of IMRT and imaging aspects of commissioning are not included in this investigation.

## II. MATERIALS AND METHODS

The Versa HD is a digital linac capable of delivering 6 MV, 6 MV FFF, 10 MV, 10 MV FFF, and 18 MV photon beams, as well as electron beams of 4, 6, 8, 9, 12, and 15 MeV. The maximum field size is $40\times 40\;{\text{cm}}^{2}$, defined by a pair of sculpted diaphragms mounted orthogonal to the multileaf collimator (MLC). The MLCs replace the jaws in the orthogonal direction and there are no backup jaws or diaphragms. The 80‐pair interdigitating MLCs have a projected leaf width of 5 mm at the isocenter over all leaves. The tungsten MLCs in the Agility collimator (Elekta, Stockholm, Sweden) are 9 cm thick and have a leaf speed of 3.5 cm/s. The carriage can travel up to 3 cm/s giving a maximum MLC speed of 6.5 cm/s. The Rubicon optical tracking system (Elekta) provides for accurate positioning of the leaves.^(1)^ The MLCs have a small tongue‐and‐groove interleaf gap, less than 0.1 mm, and are defocused from the source in order to minimize the interleaf leakage. The Agility collimator has a primary collimator speed of 9 cm/s and an isocenter clearance of 45 cm.^(2)^ Unless stated otherwise, all the measurements were taken at gantry and collimator angle of 0° in the International Electrotechnical Commission (IEC) 1217 specifications.

Pinnacle Treatment Planning System (TPS) (Pinnacle ver. 9.8, Philips Healthcare, Eindhoven, Netherlands) was used to model the photon energies using the collapsed‐cone convolution.^3^, ^4^ and electron energies using the Hogstrom pencil‐beam electron algorithms,^(5)^ using the data collected. The commissioning data were acquired at 100 cm source‐to‐surface distance (SSD) for Pinnacle TPS commissioning, as specified in the commissioning manual.^(6)^

Beam data acquisition for commissioning was based on the recommendations of AAPM TG‐106^(7)^ for appropriate detector selection, measurement techniques, etc. Measurements were made using a PTW MP3‐M water tank (PTW, Freiburg, Germany) with a scanning range of 50 × 50 × 40 cm^3^. PTW's TRUFIX system was used to place the chamber at the vertical level of the linac isocenter after taking into account the shift for the chamber effective point of measurement (EPOM). Prior to acquisition, a radiation beam center check was performed to position the chamber along the central axis (CAX) of radiation in the horizontal plane. Photon PDD measurements were made along the CAX using a PTW Semiflex 31010 chamber with a 0.125 cc active volume for both ionization field and reference. The PDD data were reacquired with PTW Diode $P(\text{active}\;\text{volume}=0.03\;{\text{mm}}^{3})$. The in‐plane and cross‐plane photon profile scans were acquired using PTW Semiflex 31010 chamber and PTW Diode P. The acquisition sampling time was set to 0.3 s for PTW Semiflex and 0.6 s for PTW Diode P.

All the scanned PDD and profile scans were processed using PTW's MEPHYSTO mc2 navigation software. The PDD data were smoothed by a least‐squares algorithm, interpolated to 0.2 mm spacing and normalized to 100% by the values at the depth of maximum dose ($d_{\text{max}}$). The profile scans were smoothed by least‐squares, interpolated to 0.2 mm spacing, and made symmetric after correcting for any positional deviation of the CAX. The beam profiles were normalized to 100% of the values at the CAX. The unprocessed and processed PDD and profile data were compared to ensure that the shapes remained consistent.

### A. Mechanical tests

As part of the mechanical checks of the linac during commissioning, the coincidence of light field and digital readout was performed by aligning graph paper at 100 cm SSD to the crosshairs. The tolerance for field size is 2 mm for symmetric jaws and 1 mm for individual asymmetric jaw setting, as per TG‐142 recommendations.^(8)^

Coincidence between mechanical front pointer and the optical distance indicator was measured at several SSDs in the range between 85 cm and 100 cm. TG‐142 recommended tolerances for the optical distance indicator is 1 mm. With a resolution of 1 cm, the front pointer has limited usage in the mechanical QAs.

Tabletop sag due to weight was tested by placing a 50 lb load at the end of fully extended couch. The tabletop sag can be measured by using the lateral wall‐mounted horizontal lasers as a reference point, upon verification of the lasers to within 2 mm tolerance at the isocenter. The sag in the couch extension was measured by placing a 50 lb weight in the center of the couch extension with the gantry angle at 0°. The sag in the center of the couch extension was measured by taking the difference in the optical distance indicator readings. TG‐142 and vendor‐specific tolerance for table top and couch extension sags are 2 mm and 10 mm, respectively.

The congruence between light field and radiation field is measured by placing a Gafchromic film (EBT2) (International Specialty Products, Wayne, NJ) at the isocenter perpendicular to the beam axis. The edges of the field light were marked on the film and then irradiated with opaque markers placed at the field boundaries. TG‐142 recommended tolerance is 2 mm.

The asymmetric jaw test was performed by exposing a Gafchromic film in two, complimentary beam split exposures. The collimator and gantry were fixed at 0° each and the film at 100 cm SSD. The tolerance is 2 mm for the entire field, as per TG‐142.

### B. Radiation/mechanical isocentricity

The coincidence of radiation isocenter with the mechanical isocenters of the gantry, collimator and couch was estimated using star shot analysis. A Gafchromic film (EBT2 film, Kodak, Rochester, NY) was exposed to five to six nonoverlapping fields of $0.5\times 20\;{\text{cm}}^{2}$ defined by the secondary collimator and MLCs, respectively, using 200 monitor units (MU). The process was repeated for various gantry, collimator, and couch angles. The film was scanned with a Vidar scanner (Vidar Systems Corp., Herndon, VA) following recommendations given in TG‐55.^(9)^ Star shot analysis was performed using RIT software (ver 6.1, Radiological Imaging Technology Inc., Colorado Springs, CO). The tolerance for radiation to mechanical isocentricity test is 2 mm diameter, as specified in TG‐142.

### C. Photon characterization — PDDs, profiles

The photon PDDs and profiles were acquired as specified in the manual for Pinnacle commissioning (Pinnacle^3^ Physics Reference Guide). PDD data were measured for square field sizes — 1 cm, 2 cm, 3 cm, 4 cm, 5 cm, 10 cm, 12 cm, 15 cm, 20 cm, 25 cm, 30 cm, and 40 cm per side, respectively. In‐plane and cross‐plane profile scans were acquired for the above‐mentioned fields at depths of $d_{\text{max}}$, 5 cm, 10 cm, and 20 cm.

The raw profile scans were processed by application of a smoothing filter and interpolated in steps of 0.2 mm using the MEPHYSTO mc2 software (PTW). Flatness, symmetry, horn, and penumbra were defined within the central 80% of the full width at half maximum (FWHM) of the processed profile.^(10)^ Within the specified region, flatness is defined as the maximum ratio between any two data points ($100\times D_{\text{max}}/D_{\text{min}}$), while symmetry is defined as the maximum ratio between two symmetric data points (100 × D_(x)_ / D_(‐x)_)_max_. Penumbra is defined as the spatial distance between the 80% and 20% of the CAX value in the profile scan of a flattened beam.

For the FFF beams, customization of PDDs to match with the flattened counterpart at 10 cm depth was performed as a part of acceptance testing. For the FFF beams, the penumbra normalization technique was adhered to.^11^, ^12^ The in‐plane and cross‐plane beam profiles of FFF beams were normalized to the FFF beam of the largest field size, $40\times 40\;{\text{cm}}^{2}$. Flatness, symmetry, and penumbra were estimated on the normalized profile using the above‐mentioned formulae.

### D. Photon characterization — output factors

Head scatter factors ($S_{c}$) were measured using a PTW Semiflex 31010 chamber suspended in air. Charged particle equilibrium was provided by brass buildup caps of sufficient wall thickness as compared to the range of contaminant electrons originating in the accelerator head.^13^, ^14^ Field sizes ranging from 5 cm × 5 cm^2^ up to $40\times 40\;{\text{cm}}^{2}$ were studied for the five photon energies with the chamber positioned at $D_{\text{max}}$.

Photon output factors ($S_{\text{cp}}$) were measured using a PTW Diode P for field sizes ranging from $1\times 1\;{\text{cm}}^{2}$ to $5\times 5\;{\text{cm}}^{2}$ and using a PTW Semiflex 31010 chamber from $3\times 3\;{\text{cm}}^{2}$ up to $40\times 40\;{\text{cm}}^{2}$. The setup used is 100 cm SSD, dosimeter at 10 cm depth, 100 MUs delivered at the maximum clinical dose rate. The average of three data point measurements was used to reduce any errors. A “daisy chaining” approach was utilized in estimation of the output factor and the readings of the two chambers were normalized to a $4\times 4\;{\text{cm}}^{2}$ field size.^(15)^

### E. Universal wedge — relative wedge factors

Elekta Versa HD is equipped with a 60° universal wedge mounted in the gantry head that moves in to yield an effective wedge angle when combined with an open photon field.^(16)^ The largest field size for a wedged field is $30\times 40\;{\text{cm}}^{2}$. The 30 cm side of the wedged field is defined by the secondary collimators. The wedge transmission factor is defined as the ratio of dose measured with the wedge to the measurement of the open beam for a $10\times 10\;{\text{cm}}^{2}$ field at 10 cm depth in a 100 cm SSD setup. PDD curves, as well as in‐plane and cross‐plane profiles, were measured for a range of field sizes (5 × 5, 10 × 10, 15 × 15, 20 × 20, 30 × 30, and $30\times 40\;{\text{cm}}^{2}$) at 100 cm SSD. Dose measurement for the above mentioned field sizes were normalized to a $10\times 10\;{\text{cm}}^{2}$ wedged field for the estimation of relative wedge factor.

### F. MLC characterization

The MLC transmission measurement was performed in the PTW MP3‐M water tank using a calibrated PTW Semiflex 31013 chamber (active volume=0.03 cc). The gantry was set to 0°, and collimator was set at 900 and the tank was set up at 100 cm SSD. The chamber was placed at a depth of $d_{\text{max}}$ with the long axis along the long axis of the MLC and the direction of the chamber motion across the leaf bank. An open field measurement was made on the CAX of a $10\times 10\;{\text{cm}}^{2}$ field at the depth of $D_{\text{max}}$. A cross‐plane profile scan was acquired with the chamber in an identical setup, but with the MLCs closed at a distance of 15 cm away from the CAX.^(17)^ The profile was measured with a step size of 1 mm using a 2 s acquisition time. The leaf transmission factor was estimated for each of the energies by the ratio of the maximum closed‐field reading to the open‐field reading at the CAX. IEC recommended maximum transmission for the MLCs is 1%.^(18)^

MLC spoke shot was performed with a Gafchromic film using five to six nonoverlapping fields of $0.5\times 20\;{\text{cm}}^{2}$ defined by the MLCs and the secondary collimator, respectively, using 200 MUs. The process is repeated for all the photon energies. Analysis was performed using RIT software and the acceptable tolerance is 2 mm diameter.^(8)^

### G. Electron characterization — PDDs, profiles, output

Elekta Versa HD has 4, 6, 8, 9, 12, and 15 MeV electron energies of which only 6, 9, 12, and 15 MeV were commissioned for clinical usage. The electron beam measurements were made for various field sizes and electron applicators required in the Pinnacle commissioning manual^(6)^ using a PTW Semiflex 31010 ionization chamber on the PTW MP3‐M water tank at 100 cm SSD. The acquired percent depth ionization data were converted to PDD using the stopping power ratio in the MEPHYSTO mc2 software. The practical range ($R_{p}$) was estimated from the PDD data using the largest electron applicator ($20\times 20\;{\text{cm}}^{2}$) for each of the electron energies. Likewise, the depths of 100%, 90%, 80%, 70%, and 50% ionization levels ($d_{\text{max}},\;R_{90},\;R_{80},\;R_{70}$, and $R_{50}$, respectively) were measured using the $10\times 10\;{\text{cm}}^{2}$ electron applicator. Besides $R_{p}$, electron beam data were characterized by the depths of 100%, 90%, 80%, and 50% dose levels denoted as $d_{\text{max}},\;D_{90},\;D_{80}$, and $D_{50}$, respectively. Other electron PDD parameters include the surface dose ($D_{s}$) measured at 0.5 mm depth on the CAX, and X‐ray background dose ($D_{x}$) measured by extrapolating the Bremsstrahlung tail to $R_{p}$.

The electron profile scans were performed at depths of $0.5\times R_{90},\;R_{90},\;R_{70}R_{50}$, and $R_{P}+2\;\text{cm}$ for each of the electron applicators. The raw electron profile data were processed by application of smoothing filter and interpolated in steps of 0.2 mm using the MEPHYSTO mc2 software. Flatness, symmetry, and penumbra of electron beam profile were defined identical to the photon beam profile.

The electron beam quality specifier was determined from $R_{50}$ measured using the water tank setup at 100 cm SSD using the $10\times 10\;{\text{cm}}^{2}$ electron applicator, as specified in TG‐51.^(19)^ The outputs of the electron energies were calibrated using a calibrated PTW Semiflex 31013 chamber. The mean electron incident energy $E_{0}$ was defined in TG‐25 as $E_{0}(\text{MeV})=2.33\times R_{50}(\text{cm})$.^(20)^ In addition, the outputs of the electron energies < 10 Mev, namely, 6 and 9 MeV, were verified using a PTW Roos plane‐parallel ionization chamber N34001 (active volume=0.35 cc) by the cross‐calibration procedure at the highest available electron energy of 15 MeV, as specified in TG‐51.^(19)^

### H. Electron cone factors

The electron cone factors were measured using the PTW Semiflex 31010 chamber positioned at a depth of $d_{\text{max}}$, for SSDs of 100 cm, 105 cm, and 110 cm normalized to the reading from using a $10\times 10\;{\text{cm}}^{2}$ electron applicator. The cone factors were evaluated for the four electron energies (6, 9, 12, and 15 MeV) using the four electron applicators ($6\times 6\;{\text{cm}}^{2}$ to $20\times 20\;{\text{cm}}^{2}$) for a range of square field sizes. The cutout field sizes made from Cerrobend included 2, 3, 4, 6, 8, 10 cm, and the maximum field size.

## III. RESULTS

### A. Mechanical tests

Using a level placed on the accessory face of the gantry, the largest deviation in digital readout of the gantry and collimator angles was recorded to be 0.2°, which is lower than the 0.5° tolerance. The couch was moved along the three axes by specified increments and the largest deviation in digital readout was recorded to be 0.8 mm which is less than the 1 mm tolerance. The lateral and sagittal lasers were verified to be well within the 1 mm tolerance. The vault does not have a ceiling or back‐pointer laser. The largest deviation in the test between light field and digital readout for field sizes from $5\times 5\;{\text{cm}}^{2}$ up to $40\times 40\;{\text{cm}}^{2}$ was estimated to be 1 mm against the 2 mm tolerance. The optical distance indicator was verified against the mechanical front pointer for distances from 85 cm to 100 cm and the largest deviation was recorded at 0.8 mm which is less than the 1 mm tolerance. The coincidence between light field and radiation field was estimated be within 1 mm for symmetric jaws and 0.5 mm for asymmetric jaw settings.

### B. Radiation/mechanical isocentricity

Isocentricity of gantry, collimator, and couch measured by star shot analysis using Gafchromic film and the RIT software was 1 mm, 0.7 mm and 0.7 mm diameter, respectively.

### C. Photon characterization — PDDs, profiles

**Figure 1:**
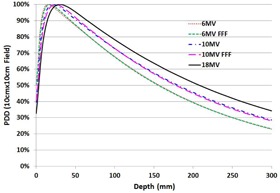
PDD curves of 6 MV, 6 MV FFF, 10 MV, 10 MV FFF, and 18 MV photon beams of a $10\times 10\;{\text{cm}}^{2}$ field at 100 cm SSD.

Figure 1 shows the PDD of $10\times 10\;{\text{cm}}^{2}$ field acquired at 100 cm SSD using PTW Semiflex 31010 chamber. Table 1 summarizes the photon beam parameters including $d_{\text{max}}$, PDD at 5, 10, and 20 cm depths for a $10\times 10\;{\text{cm}}^{2}$ field of the five photon energies. The values of $d_{\text{max}}$ of the FFF beams were deeper by 3 mm than the flattened photon beams for the $10\times 10\;{\text{cm}}^{2}$ field. While the values of PDD of the FFF beams were marginally higher than the flattened photon beams at 5 cm depth, the PDD values at 20 cm depth of the FFF beams were slightly lower than the flattened photon beams. The dose ratio in 20 cm and 5 cm depth in water (D_20_/D_5_) was specified in Table 1. While the values of D_20_/D_5_ increase with energy, there is a negligible decrease in going from a flattened beam to the FFF counterpart. The $P_{\text{ion}}$ and $P_{\text{pol}}$ values of the FFF beams are almost identical to the flattened counterparts. The PDD curve acquired using the PTW Diode P agree closely with that using PTW 31010 and the mean differences for a $10\times 10\;{\text{cm}}^{2}$ field are within 0.3%.

**Table 1:** Photon beam parameters of $d_{\text{max}}$, PDD at 5, 10, and 20 cm depths, D_20_/D_5_ ratio as well as $P_{\text{ion}},\;P_{\text{pol}}$ values of a $10\times 10\;{\text{cm}}^{2}$ field of the five photon energies.

*Energy (MV)*	$d_{\text{max}}\;(\text{cm})$	*PDD (5 cm)*	*PDD (10 cm)*	*PDD (20 cm)*	$D_{20}/D_{5}$	$P_{\text{ion}}$	$P_{\text{pol}}$
6	1.5	87	67.8	39.5	0.45	1.003	0.999
6 FFF	1.8	87.4	67.6	39.3	0.45	1.005	1.000
10	2.1	90.8	72.8	45.7	0.50	1.003	1.000
10 FFF	2.4	91.9	72.9	45.0	0.49	1.006	0.998
18	3.0	95.4	78.3	51.7	0.54	1.006	0.999

**Figure 2:**
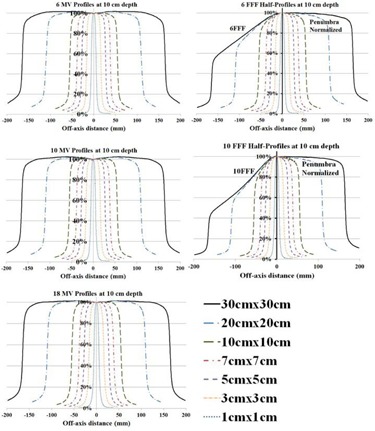
In‐plane profile scans for 6 MV, 6 MV FFF, 10 MV, 10 MV FFF, and 18 MV photon beams of square field sizes 1, 3, 5, 7, 10, 20 and 30 cm measured at a depth of 10 cm. For FFF beams, the unflattened and the normalized half‐profiles are displayed.

Figure 2 shows the inplane profile scans of 1, 3, 5, 7, 10, 20, and 30 cm square fields acquired at 100 cm SSD, 10 cm depth and a collimator angle of 0° for the five photon energies using PTW Diode P. In‐plane and cross‐plane photon profile characteristics including flatness, symmetry, and the average of left and right penumbra values, are summarized for a $10\times 10\;{\text{cm}}^{2}$ field in Table 2. While symmetry values of the FFF beams were almost identical to the flattened counterparts, flatness values were higher by 4.4% ± 0.5% for the $10\times 10\;{\text{cm}}^{2}$ field. For all energies and field sizes, the average penumbra value measured in the cross‐plane scan was higher than the average in‐plane penumbra values. For the $10\times 10\;{\text{cm}}^{2}$ field size, average cross‐plane penumbra values were higher than the average in‐plane penumbra values by 1.9 ± 0.2 mm. A two‐tailed Student's *t*‐test on the cross‐plane vs. in‐plane penumbra values revealed the presence of significant differences (p‐value <0.001).

Beam profiles measured using PTW 31010 chamber had almost identical flatness, symmetry values. However, the penumbra values of in‐plane and cross‐plane profiles of a $10\times 10\;{\text{cm}}^{2}$ field measured at 10 cm depth were larger by 2.5 mm and 1.5 mm, respectively.

**Table 2:** Flatness (%), symmetry (%), and penumbra (mm) measured from the in‐plane and cross‐plane profile scans of a $10\times 10\;{\text{cm}}^{2}$ field measured at a depth of 10 cm. Note that FFF beams are penumbra‐normalized with the $40\times 40\;{\text{cm}}^{2}$ field.

	*Flatness (%)*		*Symmetry (%)*		*Average Penumbra (mm)*	
*Energy (MV)*	*In‐plane*	*Cross‐plane*	*In‐plane*	*Cross‐plane*	*In‐plane*	*Cross‐plane*
6	101.9	102.0	100.5	100.5	5.5	7.6
6 FFF	105.7	106.5	100.8	101.1	5.1	7.2
10	101.6	102.2	100.6	100.7	5.6	8.1
10 FFF	106.7	106.8	100.9	101.7	5.8	7.3
18	102.0	102.2	100.4	100.5	6.7	8.0

### D. Photon characterization — output factors

**Figure 3:**
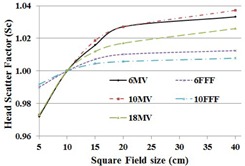
Head scatter factor ($S_{c}$) for square field sizes from 5 cm up to 40 cm for the five photon energies.

The head scatter factor was measured using a PTW Semiflex 31010 chamber with brass buildup caps of sufficient thickness for the five photon energies for field sizes from $3\times 3\;{\text{cm}}^{2}$ up to $40\times 40\;{\text{cm}}^{2}$. The head scatter factors were normalized to the respective $10\times 10\;{\text{cm}}^{2}$ field reading in each case. The head scatter factor is shown in Fig. 3. The $S_{c}$ values range from 0.97 to 1.03 for 6 MV, 0.99 to 1.01 for 6 FFF, 0.97 to 1.04 for 10 MV, 0.99 to 1.01 for 10 FFF, and 0.97 to 1.03 for 18 MV. The head scatter factor of FFF beam was lower relative to the flattened beams for field sizes larger than $10\times 10\;{\text{cm}}^{2}$ but higher for field sizes smaller than $10\times 10\;{\text{cm}}^{2}$. The ranges of output factors were smaller by 63.2% in 6 FFF in comparison with 6 MV and 75% in 10 FFF in comparison with the 10 MV beam.

**Figure 4:**
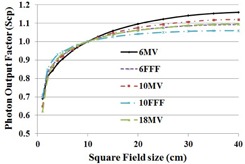
Photon output factor ($S_{\text{cp}}$) for square field sizes from 1 cm up to 40 cm for the five photon energies.

The photon output factor ($S_{\text{cp}}$) is shown in Fig. 4 for square field sizes from 1 cm up to 40 cm. The output factors ranged between 0.69 and 1.16 for 6 MV, 0.7 and 1.09 for 6 FFF, 0.65 and 1.12 for 10 MV, 0.7 and 1.06 for 10 FFF, 0.6 and 1.09 for 18 MV. The output factor of FFF beams was lower relative to the flattened beams for field sizes larger than $10\times 10\;{\text{cm}}^{2}$ but higher for field sizes smaller than $10\times 10\;{\text{cm}}^{2}$. The ranges of output factors were smaller by 18.2% in 6 FFF in comparison with 6 MV and 23.1% in 10 FFF in comparison with 10 MV beam.

### E. Universal wedge — relative wedge factors

The wedge transmission factor ranged from 0.22 to 0.31 for 6 MV, 0.24 to 0.31 for 10 MV, and 0.23 to 0.29 for 18 MV for the various field sizes. When averaged across field sizes, the transmission factors were 0.26, 0.27, and 0.26 for 6, 10, and 18 MV photon beams, respectively.

The relative wedge factors were measured at a depth of 10 cm with a SSD of 100 cm for a range of field sizes and normalized to the $10\times 10\;{\text{cm}}^{2}$ reading. The measured relative wedge factors ranged between 0.87 and 1.22 for 6 MV, between 0.88 and 1.15 for 10 MV, and between 0.9 and 1.17 for 18 MV photon beams, as tabulated in Table 3. The range of relative wedge factors decreases with increasing energy of the photon beam, but the differences are statistically insignificant with p‐value >0.1 in a two‐tailed paired Student's *t*‐test.

**Table 3:** Relative wedge factors for field sizes $5\times 5\;{\text{cm}}^{2}$ up to $30\times 40\;{\text{cm}}^{2}$ for 6, 10, and 18 MV beams.

	*Field Size (cm^2^)*					
*Energy (MV)*	*5*×*5*	*10*×*10*	*15*×*15*	*20*×*20*	*30*×*30*	*30*×*40*
6	0.87	1.00	1.07	1.17	1.20	1.22
10	0.88	1.00	1.04	1.07	1.15	1.15
18	0.9	1.00	1.07	1.12	1.16	1.17

### F. MLC characterization

MLC transmission was measured using the PTW Semiflex 31013 chamber at 10 cm depth in the PTW MP3‐M water tank set at 100 cm SSD. The average transmission for a 6 MV, 10 MV, and 18 MV beams were estimated to be 0.5%, 0.6%, and 0.6%, respectively.

MLC spoke shot analysis was performed using Gafchromic film. The analysis performed in RIT revealed isocentricity of 0.2 mm radius for the 6 MV photon beam.

### G. Electron characterization — PDDs, profiles, output

**Figure 5:**
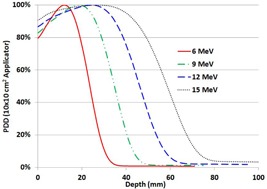
PDD of 6, 9, 12, and 15 MeV electron energies for the $10\times 10\;{\text{cm}}^{2}$ electron applicator without a custom cutout at 100 cm SSD.

Shown in Fig. 5 is the electron PDD of all energies acquired using the $10\times 10\;{\text{cm}}^{2}$ electron applicator. The PDD parameters of $d_{\text{max}},\;D_{90},\;D_{80},\;D_{50},\;R_{p},\;R_{50},\;E_{0},\;D_{s}$, and $D_{x}$ for the 6, 9, 12, and 15 MeV electron beams are mentioned in Table 4 for the $10\times 10\;{\text{cm}}^{2}$ electron applicator without a custom cutout.

**Figure 6:**
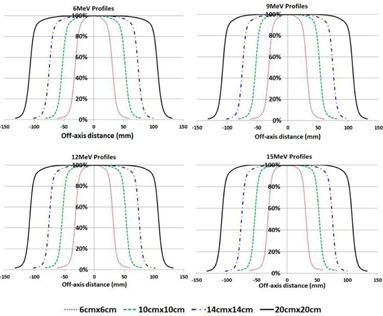
Cross‐plane profile scans of 6, 9, 12, and 15 MeV electron beams acquired for the maximum field sizes of the 6×6, 10×10, 14×14, and $20\times 20\;{\text{cm}}^{2}$ cutouts and the corresponding electron applicators at 100 cm SSD at a depth of $0.5\times R_{90}$.

Figure 6 shows the cross‐plane profile scans acquired using the maximum field sizes on the four applicators at 100 cm SSD at a depth of $0.5\times R_{90}$, as required in the Pinnacle commissioning manual.^(6)^ Electron beam profile characteristics including flatness, symmetry, and the average of left and right penumbra values for the $20\times 20\;{\text{cm}}^{2}$ applicator acquired at a depth of $0.5\times R_{90}$ at 100 cm SSD are summarized in Table 5.

The output of the electron energies calibrated using a calibrated PTW Semiflex 31013 chamber were verified with a PTW Roos chamber and the differences were estimated to be 0.4% and −0.2% for 6 and 9 MeV electron energies, respectively.

**Table 4:** Electron beam parameters of $d_{\text{max}},\;D_{90},\;D_{80},\;D_{50},\;R_{p},\;R_{50},\;E_{0},\;D_{s}$, and $D_{x}$ acquired at 100 cm SSD for the $10\times 10\;{\text{cm}}^{2}$ electron applicator without any custom cutout.

*Energy (MeV)*	$d_{\text{max}}(cm)$	$D_{90}(cm)$	$D_{80}(cm)$	$D_{50}(cm)$	$R_{p}(cm)$	$R_{50}(cm)$	$E_{0}(Mev)$	$D_{s}(\%)$	$D_{x}(\%)$
6	1.2	1.7	1.9	2.4	3.0	2.3	5.4	79.8	0.9
9	1.8	2.6	2.9	3.4	4.3	3.4	8.0	83.5	1.3
12	2.4	3.5	3.8	4.6	5.6	4.5	10.6	87.0	2.2
15	2.6	4.4	4.9	5.8	7.1	5.8	13.4	91.0	3.7

**Table 5:** Flatness (%), symmetry (%), and average penumbra (mm) measured from the in‐plane and cross‐plane profile scans for the $20\times 20\;{\text{cm}}^{2}$ electron applicator measured at a depth of $0.5\times R_{90}$.

		*Flatness (%)*		*Symmetry (%)*		*Average Penumbra (mm)*	
*Energy (MV)*	*Depth (mm)*	*In‐plane*	*Cross‐plane*	*In‐plane*	*Cross‐plane*	*In‐plane*	*Cross‐plane*
6	9.5	104.5	104.4	100.6	100.4	11.2	10.9
9	14.3	104.8	104.2	100.9	100.5	10.4	10.0
12	18.9	104.2	104.1	100.6	100.7	10.1	9.9
15	24	103.8	104.6	100.2	101.5	10.4	10.1

### H. Electron cone factors

**Figure 7:**
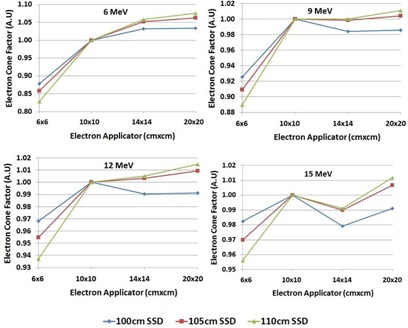
Electron cone factors for 6, 9, 12, and 15 MeV electron beams using 6×6, 10×10, 14×14, and $20\times 20\;{\text{cm}}^{2}$ electron applicators at 100, 105, and 110 cm SSD.

The cone factors for the four electron energies for SSDs of 100 cm, 105 cm, and 110 cm are shown in Fig. 7.

## IV. DISCUSSION

The overall experience in accepting and commissioning a linac can be a daunting task for any department. That difficulty is further compounded when little to no literature is available to a department for quick reference or ideas. It is the goal of this paper to aid others in commissioning a Versa HD linac. The values acquired could serve as a reference, and our procedures could aid in developing a program for future testing. The experience was one that required a significant amount of time, some of which was spent on attempting to determine the best approach to commissioning the system. This paper could potentially streamline the commissioning of a Versa HD, thus saving time and money.

The measured PDDs and beam profiles of the flattened and the FFF beams are consistent with the previous studies on Varian TrueBeam.^(21)^ The $d_{\text{max}}$ of FFF beams are deeper than the flattened beams by 3 mm for a $10\times 10\;{\text{cm}}^{2}$ field. The FFF beams also show steeper dose falloffs at depths greater than $d_{\text{max}}$ when compared to the flattened beams. This behavior can be quantified using the ratio of depth doses at 20 cm and 10 cm depths ($D_{20}$ / D_10_). The differences in $D_{20}$ / $D_{10}$ between the FFF and flattened beams were slightly more pronounced for 10 MV photon beam than for 6 MV, which is in agreement with Kragl et al.^(22)^ Removal of the flattening filter softens the energy spectra and changes the dosimetric characteristics of high‐energy photon beams.

After penumbra normalization of the FFF beam profiles, the symmetry values are almost identical while the flatness values are higher than the flattened counterparts. Over all the photon energies commissioned, the cross‐plane penumbra values defined along the MLC direction are slightly larger than the in‐plane penumbra values defined along the jaw direction by about 2 mm. The primary cause of the difference is the higher transmission through the rounded leaf ends with geometric penumbra playing a lesser role. Our data also suggest that there is an insignificant difference in the penumbra width between the FFF and the flattened beams. High‐resolution diodes and small‐volume chambers are desirable in measuring penumbra, scatter factor, and small field dosimetry. The issues with small field dosimetry include lack of lateral charged particle equilibrium, differences in detector size, and the dimensions of the radiation field.^(23)^ Overall, close agreement was observed in the ionization recombination and polarity correction between the flattened and FFF photon fields, which agrees with a Varian TrueBeam study.^(24)^ The head scatter factor and output factor of flattened beams were higher relative to the FFF beams for field sizes larger than $10\times 10\;{\text{cm}}^{2}$ but lower for field sizes smaller than $10\times 10\;{\text{cm}}^{2}$. The range of head scatter factor, output factor, and relative wedge factor values are smaller in FFF beams than the flattened counterparts, which agrees with the observation made in other studies.^25^, ^26^ Initially, the MLC transmission test was performed using a Gafchromic film that gave incorrect transmission values. The test was repeated using an ionization chamber in a water tank setup, which yielded MLC transmission values in agreement with Thompson et al.^(17)^ Characterization of electron beam PDD, profiles, and cone factors agree with a TrueBeam study.^(24)^ It is worth mentioning here that the size of an electron cutout is specified at 95 cm from the source and not at 100 cm.

## V. CONCLUSIONS

The commissioning data of the Versa HD linac, which included the percent depth dose, beam profiles, output factor, and other dosimetric data, have been measured, analyzed, and characterized systematically. Commissioning data gave valuable insights into accurate beam modeling, which determines treatment outcome and patient safety. The commissioning data may help other institutions embarking on Versa HD commissioning.
